# Economic evidence of clinical decision support systems in mental health: A systematic literature review

**DOI:** 10.1177/20552076241256511

**Published:** 2024-05-24

**Authors:** Line Stien, Carolyn Clausen, Inna Feldman, Bennett Leventhal, Roman Koposov, Kaban Koochakpour, Øystein Nytrø, Odd Sverre Westbye, Dipendra Pant, Thomas Brox Røst, Norbert Skokauskas

**Affiliations:** 1 Department of Mental Health, Regional Centre for Child and Youth Mental Health and Child Welfare (RKBU) Central Norway, Faculty of Medicine and Health Sciences, 8018 Norwegian University of Science and Technology, Trondheim, Norway; 2 Department of Public Health and Caring Sciences, Uppsala University, Uppsala, Sweden; 3 Department of Psychiatry and Behavioral Neuroscience, University of Chicago, Chicago, IL, USA; 4 Regional Centre for Child and Youth Mental Health and Child Welfare, Northern Norway, UiT - The Arctic University of Norway, Tromsø, Norway; 5 Department of Computer Science, 8018 Norwegian University of Science and Technology, Trondheim, Norway; 6 Department of Child and Adolescent Psychiatry, St Olavs Hospital, Trondheim, Norway

**Keywords:** Cost-effectiveness, clinical decision support, electronic health records, children, adolescent mental health

## Abstract

Mental health conditions are among the highest disease burden on society, affecting approximately 20% of children and adolescents at any point in time, with depression and anxiety being the leading causes of disability globally. To improve treatment outcomes, healthcare organizations turned to clinical decision support systems (CDSSs) that offer patient-specific diagnoses and recommendations. However, the economic impact of CDSS is limited, especially in child and adolescent mental health. This systematic literature review examined the economic impacts of CDSS implemented in mental health services. We planned to follow PRISMA reporting guidelines and found only one paper to describe health and economic outcomes. A randomized, controlled trial of 336 participants found that 60% of the intervention group and 32% of the control group achieved symptom reduction, i.e. a 50% decrease as per the Symptom Checklist-90-Revised (SCL-90-R), a method to evaluate psychological problems and identify symptoms. Analysis of the incremental cost-effectiveness ratio found that for every 1% of patients with a successful treatment result, it added €57 per year. There are not enough studies to draw conclusions about the cost-effectiveness in a mental health context. More studies on economic evaluations of the viability of CDSS within mental healthcare have the potential to contribute to patients and the larger society.

## Health economics: evaluating mental health interventions

Mental health is a state of complete physical, psychological, and social well-being.^1,2^ According to WHO's “*Comprehensive Mental Health Action Plan 2013–2030,”* it is vital that each member state meets children's and adolescents’ needs.^3^ Mental health conditions are among the most common clinical challenges affecting approximately 20% of children and adolescents.^4,5^ Mental disorders that begin early often persist into adulthood with devastating consequences and increased medical and social services utilization.^6,7^ Depression and anxiety alone cost around $1 trillion annually in lost productivity due to sickness and unemployment.^2^ Despite this, the median budget allocation for mental health is 2% of governments’ health budgets globally.^8^ Many of these costs can be avoided by considering early assessments and facilitating access to treatments.^9^

Health economic evaluations (EEs) aim to improve decision-making and patient outcomes.^10^ Given the scarcity and intensity of resource utilization in healthcare, EEs often identify the interventions or treatments that provide the best resource use.^10^ Cost-effectiveness analysis (CEA) is a standard method “to compare treatments and preventive measures in terms of their efficiency, that is, their ability to generate health and well-being relative to the costs incurred,”^10^ [p. 71] or shortly, to identify which interventions produce the best outcomes at the *best* price. In this way, CEA “compares the costs and health effects of two or more interventions.”^10^ [p. 73] Currently, without early interventions, child and adolescent mental health conditions can develop into lifelong and unfavorable outcomes.^4^ Solutions thus require (A) innovative service systems and care and (B) a holistic economic perspective—clear options exist to accomplish these goals.

Digital tools are crucial to increasing accessibility to healthcare.^11^ In child and adolescent psychiatry, a useful strategy is providing clinicians with digital tools for decision-making for personalized prediction, diagnosis, and treatment.^7^ Healthcare organizations increasingly turn to clinical decision support systems (CDSSs), an ICT offering patient-specific advice based on guidelines, expert opinion, and/or artificial intelligence. A CDSS aims to assist clinicians and healthcare providers in analyzing patient data and using that information for formulating a diagnosis and developing a treatment plan, enabling them to improve the quality of the care their patients receive.^7,12^ Studies have shown that CDSS can improve practices, reduce medical errors, and improve adherence to recommended care standards, resulting in improved clinical practice.^12^

## Understanding the economic impact of CDSS

**Figure 1.**
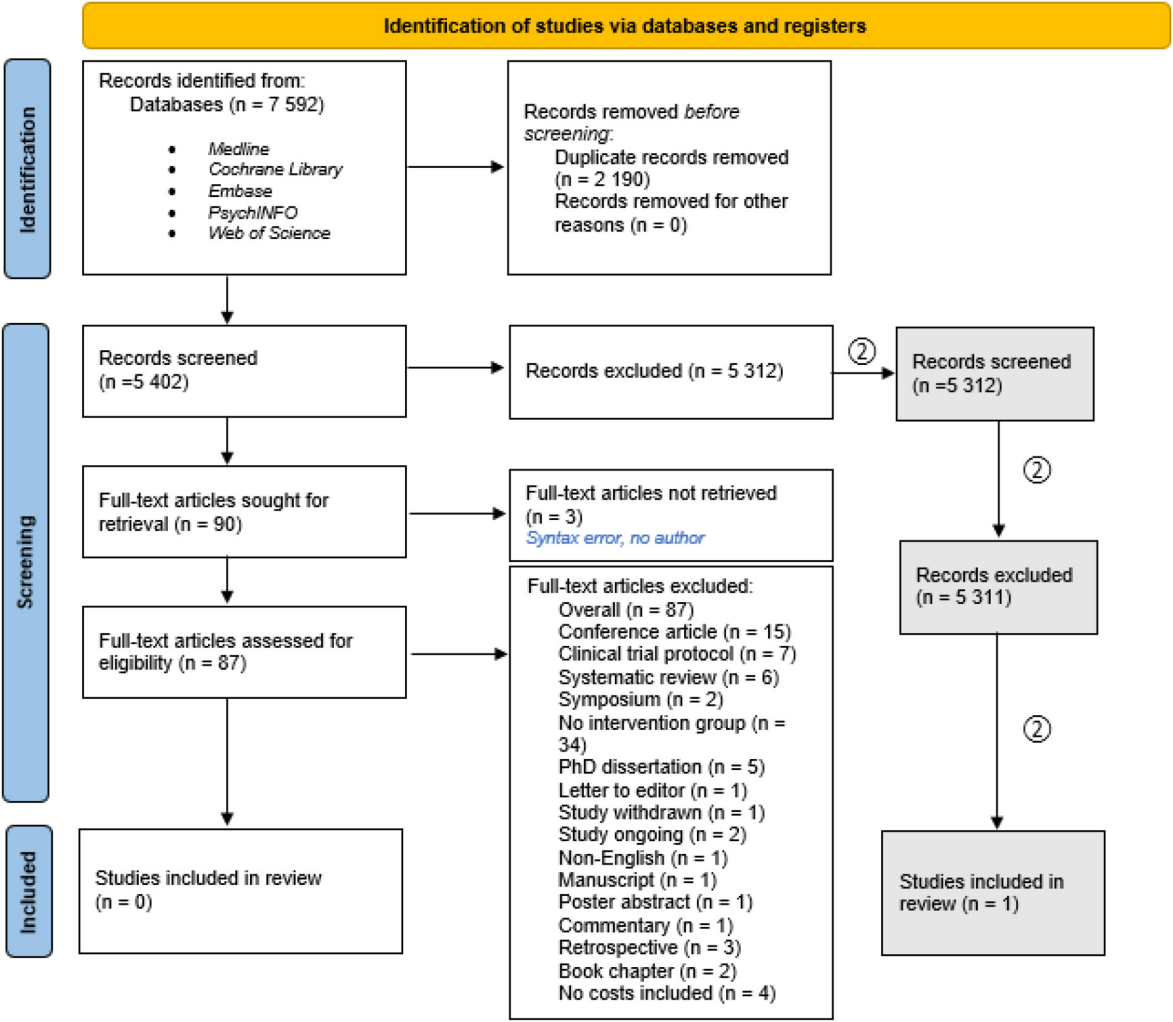
The literature review process. Based on PRISMA flowchart from Page et al. 2021.^15^

The use of CDSS in mental health is lacking and limited for children and adolescents.^13^ However, the economic impact of CDSS implementation within other medical fields varies, partially because of lacking effect measures using standardized measurement tools.^12,14^ We examined the economic evidence of CDSS implementation as a support for mental health services. To achieve that, we employed the PRISMA reporting guidelines^a, 15^ (Figure 1). The studies with a health economic focus were examined, and 87 papers were deemed eligible for full-text screening (Table 1). A second screening (no. “2” in Figure 1) was performed to locate articles on somatic illnesses.

**Table 1.** Inclusion and exclusion criteria.

Inclusion criteria	Exclusion criteria
Peer-reviewed scientific reports published in English, i.e. controlled trials cohort studies. Published between 2000 and 2022. Case–control studies and economic analyses comparing two or more options. CDSS intervention compared to standard care.	Case reports and letters. Literature reviews, systematic reviews, and commentaries. Published before 2000 Not including either CDSS or mental health linked with economics.

CDSS: clinical decision support system.

We found only one article describing some economic and health outcomes from implementing a diagnosis support an e-tool in primary healthcare.^16^ This study^16^ evaluated PsyScan, an e-tool comprised of a distress screener and a health questionnaire used by patients to get advice between general practitioner (GP) consultations (Table 2). The study was conducted in 10 multidisciplinary primary healthcare centers in Eindhoven, the Netherlands and presents a randomized, controlled trial with a 1-year follow-up. In this study, the participants received usual care from their GPs (control group (CG), n = 160) or GP consultations through PsyScan (intervention group (IG), n = 176); 60% of the IG and 32% of the CG received successful treatment results. A successful treatment result was measured by symptom reduction, i.e. a 50% decrease as per the Symptom Checklist-90-Revised (SCL-90-R), a method to evaluate psychological problems and identify symptoms. A CEA evaluated the incremental cost-effectiveness ratio (ICER) based on participants’ complete cost- and outcome data. The costs included healthcare consumption in primary and secondary care. The mean 1-year cost differed by €1135 between IG and CG. The ICER demonstrates that for every 1% of patients with a successful treatment result, the treatment costs additionally €57 yearly, compared to usual care. It was concluded that results were significant in support of PsyScan and its ability to help patients to recover; there were some, albeit modest, indications that the e-tool was cost-effective.^16^

**Table 2.** Gidding et al.'s sampling and study process.

A sample size of 336	
Upon registration at the primary care center, the patients were asked to participate in the study.	Patients were over 18 years old, Dutch-speaking, had a suspected physical problem, could consent, and could use the tool at home.
1-year pilot with measures at 3, 6, and 12 months.	

## Where are we going from here?

Our review suggests a lack of studies providing a clear roadmap for implementing CDSS in mental healthcare. This supports the argument that we need more research on CDSS's cost-effectiveness in healthcare to confirm whether it is a sound investment, both financially and in terms of clinical outcomes. While studies show that CDSS can improve effectiveness and communications,^4,12^ it is necessary to prove their cost-effectiveness, otherwise the broad implementation of CDSS might be problematic for today's healthcare system's challenging and constant budget constraints. Economic assessments provide compelling arguments for healthcare investments.^17^ EEs are critical for decision-makers and policymakers to make informed decisions and ensure sustainability in healthcare.^18^ There does not appear to be clear and consistent evidence of the cost-effectiveness of CDSS in mental health so far, except.^16^ Thus, more studies on EEs of the viability of CDSS within mental healthcare could make significant contributions.

## Notes

a. (Prospero registration number CRD42022343630)
